# (1*R*,3*S*)-Methyl 6,7-dimeth­oxy-1-(4-meth­oxy­phen­yl)-1,2,3,4-tetra­hydro­isoquinoline-3-carboxyl­ate

**DOI:** 10.1107/S1600536810044909

**Published:** 2010-11-06

**Authors:** Tricia Naicker, Thavendran Govender, Hendrik G. Kruger, Glenn E. M. Maguire

**Affiliations:** a School of Chemistry, University of KwaZulu-Natal, Durban 4000, South Africa; b School of Pharmacy and Pharmacology, University of KwaZulu-Natal, Durban 4000, South Africa

## Abstract

The title compound, C_20_H_23_NO_5_, is the third in a series of tetra­hydoisoquinoline (TIQ) compounds that are precursors to novel chiral catalysts. The N-containing six-membered ring assumes a half-boat conformation. No hydrogen bonding is observed in the crystal structure.

## Related literature

For related structures, see: Naicker *et al.* (2009 ▶, 2010 ▶); Alberach *et al.* (2004 ▶). For the synthesis of the title compound, see: Aubry *et al.* (2006 ▶).
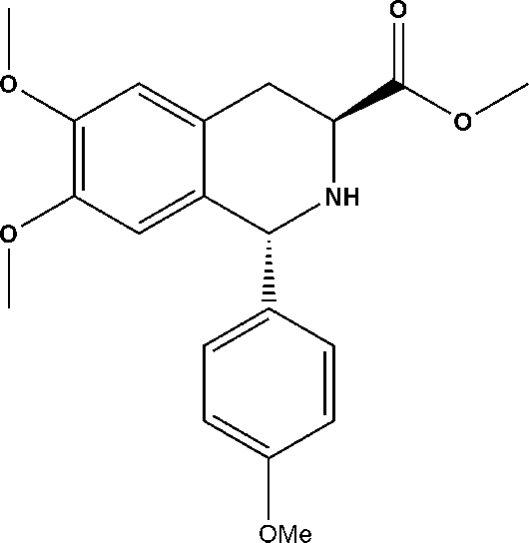


## Experimental

### 

#### Crystal data

C_20_H_23_NO_5_
*M* *_r_* = 357.39
Orthorhombic, 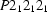

*a* = 5.3719 (7) Å
*b* = 12.1726 (14) Å
*c* = 27.021 (3) Å
*V* = 1766.9 (4) Å^3^
*Z* = 4
Mo *K*α radiation
μ = 0.10 mm^−1^
*T* = 173 K
0.20 × 0.12 × 0.12 mm

#### Data collection

Bruker Kappa DUO APEXII diffractometer
13619 measured reflections
2878 independent reflections
2538 reflections with *I* > 2σ(*I*)
*R*_int_ = 0.032

#### Refinement

*R*[*F* ^2^ > 2σ(*F* ^2^)] = 0.036
*wR*(*F* ^2^) = 0.090
*S* = 1.04
2878 reflections
239 parameters
1 restraint
H atoms treated by a mixture of independent and constrained refinement
Δρ_max_ = 0.26 e Å^−3^
Δρ_min_ = −0.18 e Å^−3^

### 

Data collection: *APEX2* (Bruker, 2006 ▶); cell refinement: *SAINT* (Bruker, 2006 ▶); data reduction: *SAINT*; program(s) used to solve structure: *SHELXS97* (Sheldrick, 2008 ▶); program(s) used to refine structure: *SHELXL97* (Sheldrick, 2008 ▶); molecular graphics: *OLEX2* (Dolomanov *et al.*, 2009 ▶); software used to prepare material for publication: *SHELXL97*.

## Supplementary Material

Crystal structure: contains datablocks I, global. DOI: 10.1107/S1600536810044909/hg2711sup1.cif

Structure factors: contains datablocks I. DOI: 10.1107/S1600536810044909/hg2711Isup2.hkl

Additional supplementary materials:  crystallographic information; 3D view; checkCIF report

## Figures and Tables

**Fig. 1.**
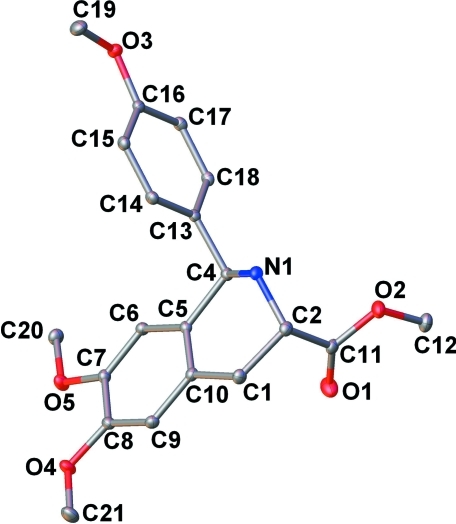
The title compound (all H atoms omitted for clarity). All non-H atoms are shown as ellipsoids with probability level of 50%.

**Fig. 2.**
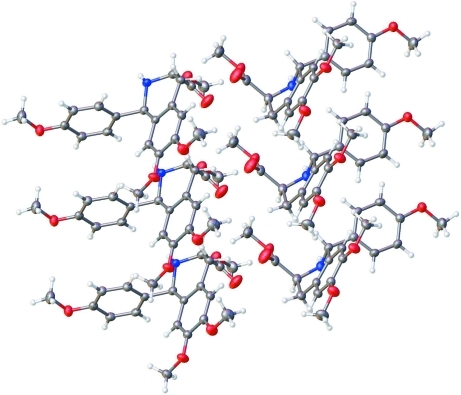
Projection viewed along [010].

## supplementary crystallographic information

### Comment

The title compound was derived from commercially available *L*-DOPA and anisaldehyde. Diastereomers formed during the first step of the synthesis were separated to yield subsequent derivatives and the title compound with the stereochemistry as illustrated in Fig. 1. The title compound is the third report in a series of molecules containing a tetrahydroisoquinoline backbone and is a precursor to one of the molecules that we previously reported ((1*R*,3*S*)-methyl 2-benzyl-6,7-dimethoxy-1-phenyl-1,2,3,4-tetrahydroisoquinoline-3-carboxylate), (Naicker *et al.*, 2009). The molecule has been reported previously and the absolute stereochemistry of the diastereomer was confirmed to be *R,S* at C4 and C2 positions respectively by proton NMR (Aubry *et al.*, 2006).

There are a number of common features found in this structure and that of the the unprotected secondary amine system. First, the *N*-containing six membered ring assumes a half boat conformation. This differs from last report for the (1*R*,3S)-2-benzyl-6,7-dimethoxy-1-phenyl-1,2,3,4 tetrahydroisoquinolin-3-yl diphenylmethanol structure (Naicker *et al.*, 2010) and previous reports by Alberach *et al.* (2004) and Aubry *et al*. (2006) where the heteroatomic ring adopted a half chair conformation. Second, given the presence of the secondary amine, ether and in this example ester functional groups, no hydrogen bonding is observed in any of the structures of this series, (see Fig. 2).

### Experimental

A solution of the Cbz protected *trans*-6,7-dimethoxy-1-(4-methoxyphenyl)-TIQ methyl ester (1.0 g, 0.21 mmol) in THF (20 ml) was added to a suspension of activated 10 wt% Pd/C (500 mg) in dry MeOH (20 ml). The mixture was connected to a hydrogen source at one atmosphere and stirred at room temperature for 1 h. Completion of the reaction was monitored through TLC in hexane/ethyl acetate (50/50, *R*_f_ = 0.6). The Pd/C was filtered through a Celite pad and washed with methanol (20 ml). The filtrate was evaporated under reduced pressure affording the crude amino ester, which was purified by column chromatography using ethyl acetate/hexane (50:50) as the eluent to yield pure title compound (0.70 g, 93%) as a yellow solid. m.p. = 392–393 K. Crystals suitable for X-ray diffraction were obtained by slow evaporation of the title compound in MeOH at room temperature.

^1^H NMR (600 MHz, CdCl_3_, d, p.p.m.): 1.58 (broad s, 1H), 2.99 (dd, 1H), 3.09 (dd, 1H), 3.60 (s, 3H), 3.66 (s, 3H) 3.67(s, 3H), 3.78 (m, 1H), 3.88 (s, 3H), 5.23 (s, 1H), 6.30(s, 1H), 6.61 (s, 1H), 6.82 (d, 2H), 7.09 (d, 2H).

IR: 2946 (w), 1700 (w), 1507 (*s*), 1223 (*vs*), 832 (*s*), 563 (w)

### Refinement

All H atoms, except H1N, were positioned geometrically with C—H distances ranging from 0.95 Å to 1.00 Å and refined as riding on their parent atoms, with *U*_iso_ (H) = 1.2–1.5*U*_eq_ (C).

### Crystal data

**Table:**

C_20_H_23_NO_5_	*F*(000) = 760
*M**_r_* = 357.39	*D*_x_ = 1.344 Mg m^−^^3^
Orthorhombic, *P*2_1_2_1_2_1_	Mo *K*α radiation, λ = 0.71073 Å
Hall symbol: P 2ac 2ab	Cell parameters from 13619 reflections
*a* = 5.3719 (7) Å	θ = 2.3–29.6°
*b* = 12.1726 (14) Å	µ = 0.10 mm^−^^1^
*c* = 27.021 (3) Å	*T* = 173 K
*V* = 1766.9 (4) Å^3^	Needle, colourless
*Z* = 4	0.20 × 0.12 × 0.12 mm

### Data collection

**Table:**

Bruker Kappa DUO APEXII diffractometer	2538 reflections with *I* > 2σ(*I*)
Radiation source: fine-focus sealed tube	*R*_int_ = 0.032
graphite	θ_max_ = 29.6°, θ_min_ = 2.3°
0.5° φ scans and ω scans	*h* = −7→7
13619 measured reflections	*k* = −16→16
2878 independent reflections	*l* = −26→37

### Refinement

**Table:**

Refinement on *F*^2^	Primary atom site location: structure-invariant direct methods
Least-squares matrix: full	Secondary atom site location: difference Fourier map
*R*[*F*^2^ > 2σ(*F*^2^)] = 0.036	Hydrogen site location: inferred from neighbouring sites
*wR*(*F*^2^) = 0.090	H atoms treated by a mixture of independent and constrained refinement
*S* = 1.04	*w* = 1/[σ^2^(*F*_o_^2^) + (0.0435*P*)^2^ + 0.3587*P*] where *P* = (*F*_o_^2^ + 2*F*_c_^2^)/3
2878 reflections	(Δ/σ)_max_ = 0.001
239 parameters	Δρ_max_ = 0.26 e Å^−^^3^
1 restraint	Δρ_min_ = −0.18 e Å^−^^3^

### Special details

**Table:**

Geometry. All e.s.d.'s (except the e.s.d. in the dihedral angle between two l.s. planes) are estimated using the full covariance matrix. The cell e.s.d.'s are taken into account individually in the estimation of e.s.d.'s in distances, angles and torsion angles; correlations between e.s.d.'s in cell parameters are only used when they are defined by crystal symmetry. An approximate (isotropic) treatment of cell e.s.d.'s is used for estimating e.s.d.'s involving l.s. planes.
Refinement. Refinement of *F*^2^ against ALL reflections. The weighted *R*-factor *wR* and goodness of fit *S* are based on *F*^2^, conventional *R*-factors *R* are based on *F*, with *F* set to zero for negative *F*^2^. The threshold expression of *F*^2^ > σ(*F*^2^) is used only for calculating *R*-factors(gt) *etc*. and is not relevant to the choice of reflections for refinement. *R*-factors based on *F*^2^ are statistically about twice as large as those based on *F*, and *R*- factors based on ALL data will be even larger.

### Fractional atomic coordinates and isotropic or equivalent isotropic displacement parameters (Å^2^)

**Table:**

	*x*	*y*	*z*	*U*_iso_*/*U*_eq_	
C1	0.3734 (3)	0.44954 (13)	0.76583 (6)	0.0498 (4)	
H1A	0.2473 (3)	0.28121 (11)	0.78678 (5)	0.0378 (3)	
H1B	0.4011 (3)	0.34146 (11)	1.09674 (5)	0.0307 (3)	
C2	0.3315 (3)	0.91415 (10)	0.85890 (5)	0.0351 (3)	
H2	0.6521 (3)	0.82927 (10)	0.91879 (5)	0.0334 (3)	
C4	0.0808 (3)	0.40422 (12)	0.87542 (5)	0.0253 (3)	
H4	0.078 (4)	0.3258 (5)	0.8806 (7)	0.031 (5)*	
C5	−0.0280 (3)	0.55050 (15)	0.81826 (7)	0.0269 (4)	
C6	−0.0184	0.5713	0.7829	0.032*	
H6	−0.1997	0.5652	0.8298	0.032*	
C7	0.0265 (3)	0.42813 (14)	0.82338 (6)	0.0264 (4)	
C8	−0.1279	0.3871	0.8141	0.032*	
C9	0.3147 (3)	0.45361 (13)	0.89317 (6)	0.0224 (3)	
H9	0.4574	0.4190	0.8752	0.027*	
C10	0.3126 (3)	0.57639 (13)	0.88213 (6)	0.0211 (3)	
C11	0.4841 (3)	0.64470 (14)	0.90671 (6)	0.0229 (3)	
C12	0.5967	0.6136	0.9299	0.027*	
H12A	0.4912 (3)	0.75648 (14)	0.89759 (6)	0.0242 (3)	
H12B	0.3187 (4)	0.80245 (13)	0.86413 (6)	0.0250 (3)	
H12C	0.1543 (3)	0.73469 (14)	0.83937 (6)	0.0253 (3)	
C13	0.0407	0.7657	0.8164	0.030*	
C14	0.1519 (3)	0.62062 (14)	0.84759 (6)	0.0231 (3)	
H14	0.2348 (4)	0.39131 (15)	0.78851 (6)	0.0276 (4)	
C15	0.4358 (4)	0.23681 (18)	0.75419 (8)	0.0383 (5)	
H15	0.4304	0.1564	0.7553	0.057*	
C16	0.6003	0.2623	0.7649	0.057*	
C17	0.4044	0.2617	0.7203	0.057*	
H17	0.3392 (3)	0.42874 (12)	0.94812 (6)	0.0216 (3)	
C18	0.5433 (3)	0.37117 (13)	0.96620 (6)	0.0239 (3)	
H18	0.6727	0.3501	0.9442	0.029*	
C19	0.5606 (3)	0.34403 (14)	1.01604 (7)	0.0256 (3)	
H19A	0.7007	0.3045	1.0280	0.031*	
H19B	0.3716 (3)	0.37499 (13)	1.04827 (6)	0.0238 (3)	
H19C	0.1682 (3)	0.43478 (13)	1.03116 (6)	0.0250 (3)	
C20	0.0406	0.4574	1.0533	0.030*	
H20A	0.1551 (3)	0.46090 (13)	0.98113 (6)	0.0245 (3)	
H20B	0.0167	0.5017	0.9692	0.029*	
H20C	0.1949 (4)	0.35863 (18)	1.12932 (7)	0.0399 (5)	
C21	0.2374	0.3319	1.1625	0.060*	
H21A	0.0496	0.3185	1.1169	0.060*	
H21B	0.1564	0.4372	1.1309	0.060*	
H21C	0.8323 (4)	0.78695 (16)	0.95244 (7)	0.0317 (4)	
N1	0.9353	0.8473	0.9650	0.047*	
H1N	0.9380	0.7335	0.9353	0.047*	
O1	0.7474	0.7510	0.9802	0.047*	
O2	0.1569 (4)	0.96322 (17)	0.82600 (8)	0.0419 (5)	
O3	0.1835	1.0429	0.8252	0.063*	
O4	−0.0125	0.9477	0.8375	0.063*	
O5	0.1797	0.9329	0.7927	0.063*	

### Atomic displacement parameters (Å^2^)

**Table:**

	*U*^11^	*U*^22^	*U*^33^	*U*^12^	*U*^13^	*U*^23^
C1	0.0537 (10)	0.0375 (8)	0.0581 (10)	−0.0089 (8)	0.0274 (9)	−0.0036 (7)
H1A	0.0463 (8)	0.0301 (7)	0.0370 (7)	−0.0025 (6)	0.0121 (7)	−0.0065 (6)
H1B	0.0360 (7)	0.0309 (6)	0.0252 (6)	0.0054 (6)	0.0012 (5)	0.0018 (5)
C2	0.0433 (8)	0.0212 (6)	0.0409 (8)	−0.0014 (6)	−0.0095 (7)	0.0042 (5)
H2	0.0349 (7)	0.0253 (6)	0.0399 (7)	−0.0042 (6)	−0.0127 (6)	−0.0019 (5)
C4	0.0265 (7)	0.0241 (7)	0.0253 (7)	−0.0065 (6)	0.0018 (6)	−0.0012 (6)
C5	0.0242 (8)	0.0296 (8)	0.0269 (8)	−0.0021 (7)	−0.0041 (7)	−0.0024 (7)
C7	0.0256 (8)	0.0276 (8)	0.0261 (8)	−0.0063 (7)	0.0012 (7)	−0.0034 (7)
C9	0.0226 (7)	0.0205 (7)	0.0242 (7)	−0.0020 (6)	0.0033 (6)	−0.0014 (6)
C10	0.0214 (7)	0.0201 (7)	0.0218 (7)	−0.0015 (6)	0.0032 (6)	−0.0004 (6)
C11	0.0221 (7)	0.0237 (7)	0.0229 (7)	0.0002 (6)	−0.0005 (6)	0.0009 (6)
H12A	0.0246 (8)	0.0236 (7)	0.0246 (8)	−0.0022 (7)	−0.0007 (7)	−0.0023 (6)
H12B	0.0292 (8)	0.0207 (7)	0.0252 (8)	−0.0003 (7)	0.0020 (7)	0.0014 (6)
H12C	0.0256 (8)	0.0268 (8)	0.0235 (8)	0.0008 (7)	−0.0023 (7)	0.0023 (6)
C14	0.0229 (7)	0.0254 (7)	0.0211 (7)	−0.0019 (7)	0.0015 (7)	−0.0027 (6)
H14	0.0302 (9)	0.0302 (9)	0.0223 (8)	−0.0047 (7)	−0.0004 (7)	−0.0057 (7)
C15	0.0400 (11)	0.0407 (11)	0.0342 (10)	0.0036 (9)	0.0033 (9)	−0.0115 (9)
H17	0.0226 (7)	0.0175 (7)	0.0248 (8)	−0.0022 (6)	0.0006 (7)	−0.0006 (6)
C18	0.0198 (7)	0.0214 (7)	0.0304 (8)	0.0000 (6)	0.0042 (7)	−0.0027 (6)
C19	0.0214 (7)	0.0225 (7)	0.0329 (9)	0.0021 (6)	−0.0017 (7)	0.0004 (7)
H19B	0.0281 (8)	0.0185 (7)	0.0247 (8)	−0.0027 (6)	−0.0004 (7)	−0.0006 (6)
H19C	0.0252 (7)	0.0220 (7)	0.0278 (8)	0.0027 (7)	0.0041 (7)	−0.0028 (6)
H20A	0.0235 (7)	0.0211 (7)	0.0289 (8)	0.0036 (7)	0.0012 (7)	0.0006 (6)
H20C	0.0469 (12)	0.0432 (11)	0.0296 (9)	0.0033 (10)	0.0082 (9)	0.0042 (8)
H21C	0.0302 (9)	0.0348 (9)	0.0300 (9)	−0.0043 (8)	−0.0062 (8)	−0.0030 (7)
O2	0.0488 (12)	0.0272 (9)	0.0498 (12)	0.0069 (10)	−0.0084 (11)	0.0058 (8)

### Geometric parameters (Å, °)

**Table:**

C1—H14	1.197 (2)	H12B—H12C	1.381 (2)
H1A—H14	1.343 (2)	H12C—C13	0.9500
H1A—C15	1.446 (2)	H12C—C14	1.406 (2)
H1B—H19B	1.381 (2)	C15—H15	0.9800
H1B—H20C	1.430 (2)	C15—C16	0.9800
C2—H12B	1.369 (2)	C15—C17	0.9800
C2—O2	1.424 (2)	H17—C18	1.390 (2)
H2—H12A	1.364 (2)	H17—H20A	1.388 (2)
H2—H21C	1.425 (2)	C18—H18	0.9500
C4—H4	0.965 (5)	C18—C19	1.390 (2)
C4—C7	1.465 (2)	C19—H19A	0.9500
C4—C9	1.473 (2)	C19—H19B	1.390 (2)
C5—C6	0.9900	H19B—H19C	1.392 (2)
C5—H6	0.9900	H19C—C20	0.9500
C5—C7	1.524 (3)	H19C—H20A	1.391 (2)
C5—C14	1.514 (2)	H20A—H20B	0.9500
C7—C8	1.0000	H20C—C21	0.9800
C7—H14	1.530 (3)	H20C—H21A	0.9800
C9—H9	1.0000	H20C—H21B	0.9800
C9—C10	1.524 (2)	H21C—N1	0.9800
C9—H17	1.521 (2)	H21C—H1N	0.9800
C10—C11	1.407 (2)	H21C—O1	0.9800
C10—C14	1.381 (2)	O2—O3	0.9800
C11—C12	0.9500	O2—O4	0.9800
C11—H12A	1.383 (2)	O2—O5	0.9800
H12A—H12B	1.410 (2)		
			
C1—H14—H1A	122.83 (18)	H12B—C2—O2	116.62 (16)
C1—H14—C7	126.64 (17)	H12B—H12C—C13	119.4
H1A—H14—C7	110.53 (15)	H12B—H12C—C14	121.27 (16)
H1A—C15—H15	109.5	H12C—H12B—H12A	119.57 (15)
H1A—C15—C16	109.5	H12C—C14—C5	118.69 (16)
H1A—C15—C17	109.5	C14—C5—C6	109.1
H1B—H19B—C19	115.50 (16)	C14—C5—H6	109.1
H1B—H19B—H19C	124.03 (16)	C14—C5—C7	112.38 (15)
H1B—H20C—C21	109.5	C14—C10—C9	121.29 (15)
H1B—H20C—H21A	109.5	C14—C10—C11	119.86 (15)
H1B—H20C—H21B	109.5	C14—H12C—C13	119.4
C2—H12B—H12A	115.30 (16)	H14—H1A—C15	115.43 (16)
C2—H12B—H12C	125.13 (16)	H14—C7—C8	107.8
C2—O2—O3	109.5	H15—C15—C16	109.5
C2—O2—O4	109.5	H15—C15—C17	109.5
C2—O2—O5	109.5	C16—C15—C17	109.5
H2—H12A—C11	125.56 (16)	H17—C9—H9	108.9
H2—H12A—H12B	115.32 (15)	H17—C9—C10	112.76 (13)
H2—H21C—N1	109.5	H17—C18—H18	119.6
H2—H21C—H1N	109.5	H17—H20A—H19C	121.60 (16)
H2—H21C—O1	109.5	H17—H20A—H20B	119.2
C4—C7—C5	108.63 (14)	C18—H17—C9	120.77 (15)
C4—C7—C8	107.8	C18—C19—H19A	120.2
C4—C7—H14	112.79 (15)	C19—C18—H17	120.87 (16)
C4—C9—H9	108.9	C19—C18—H18	119.6
C4—C9—C10	109.28 (14)	C19—H19B—H19C	120.46 (16)
C4—C9—H17	108.07 (13)	H19B—H1B—H20C	116.85 (15)
C5—C7—C8	107.8	H19B—C19—C18	119.62 (16)
C5—C7—H14	111.78 (15)	H19B—C19—H19A	120.2
C6—C5—H6	107.9	H19B—H19C—C20	120.6
C7—C4—H4	109.5 (12)	H19C—H20A—H20B	119.2
C7—C4—C9	113.65 (13)	H20A—H17—C9	120.63 (15)
C7—C5—C6	109.1	H20A—H17—C18	118.58 (15)
C7—C5—H6	109.1	H20A—H19C—H19B	118.84 (16)
C9—C4—H4	111.7 (13)	H20A—H19C—C20	120.6
C10—C9—H9	108.9	C21—H20C—H21A	109.5
C10—C11—C12	119.5	C21—H20C—H21B	109.5
C10—C14—C5	122.23 (15)	H21A—H20C—H21B	109.5
C10—C14—H12C	119.08 (16)	N1—H21C—H1N	109.5
C11—C10—C9	118.82 (15)	N1—H21C—O1	109.5
C11—H12A—H12B	119.11 (16)	H1N—H21C—O1	109.5
H12A—H2—H21C	117.63 (14)	O3—O2—O4	109.5
H12A—C11—C10	121.00 (16)	O3—O2—O5	109.5
H12A—C11—C12	119.5	O4—O2—O5	109.5
			
H1B—H19B—H19C—H20A	−177.95 (16)	C11—C10—C14—C5	177.06 (15)
C2—H12B—H12C—C14	−179.09 (17)	C11—C10—C14—H12C	−3.2 (2)
H2—H12A—H12B—C2	−2.1 (2)	C11—H12A—H12B—C2	177.27 (16)
H2—H12A—H12B—H12C	177.55 (16)	C11—H12A—H12B—H12C	−3.1 (3)
C4—C7—H14—C1	−110.1 (2)	H12A—H12B—H12C—C14	1.3 (3)
C4—C7—H14—H1A	68.92 (19)	H12B—H12C—C14—C5	−178.36 (16)
C4—C9—C10—C11	163.71 (14)	H12B—H12C—C14—C10	1.9 (3)
C4—C9—C10—C14	−18.3 (2)	C14—C5—C7—C4	43.2 (2)
C4—C9—H17—C18	121.18 (16)	C14—C5—C7—H14	−81.93 (18)
C4—C9—H17—H20A	−57.33 (19)	C14—C10—C11—H12A	1.4 (2)
C5—C7—H14—C1	12.7 (3)	C15—H1A—H14—C1	−1.9 (3)
C5—C7—H14—H1A	−168.34 (16)	C15—H1A—H14—C7	179.03 (15)
C7—C4—C9—C10	53.33 (17)	H17—C9—C10—C11	43.5 (2)
C7—C4—C9—H17	176.36 (14)	H17—C9—C10—C14	−138.55 (16)
C7—C5—C14—C10	−11.8 (2)	H17—C18—C19—H19B	−0.1 (3)
C7—C5—C14—H12C	168.42 (16)	C18—H17—H20A—H19C	−1.4 (2)
C9—C4—C7—C5	−67.42 (18)	C18—C19—H19B—H1B	178.05 (15)
C9—C4—C7—H14	57.07 (19)	C18—C19—H19B—H19C	−1.3 (2)
C9—C10—C11—H12A	179.36 (15)	C19—H19B—H19C—H20A	1.3 (2)
C9—C10—C14—C5	−0.9 (2)	H19B—H19C—H20A—H17	0.0 (3)
C9—C10—C14—H12C	178.92 (15)	H20A—H17—C18—C19	1.5 (2)
C9—H17—C18—C19	−177.06 (15)	H20C—H1B—H19B—C19	−171.63 (16)
C9—H17—H20A—H19C	177.10 (15)	H20C—H1B—H19B—H19C	7.7 (2)
C10—C9—H17—C18	−117.93 (17)	H21C—H2—H12A—C11	1.4 (3)
C10—C9—H17—H20A	63.6 (2)	H21C—H2—H12A—H12B	−179.25 (15)
C10—C11—H12A—H2	−178.92 (16)	O2—C2—H12B—H12A	−178.97 (16)
C10—C11—H12A—H12B	1.8 (3)	O2—C2—H12B—H12C	1.4 (3)
